# Fatty acid composition and phospholipid types used in infant formulas modifies the establishment of human gut bacteria in germ-free mice

**DOI:** 10.1038/s41598-017-04298-0

**Published:** 2017-06-21

**Authors:** Rikke Guldhammer Nejrup, Tine Rask Licht, Lars Ingvar Hellgren

**Affiliations:** 1 Department of Systems Biology, Technical University of Denmark, Building 224, Lyngby, DK-2800 Denmark; 2 National Food Institute, Technical University of Denmark, Lyngby, Denmark

## Abstract

Human milk fat contains high concentrations of medium-chained fatty acids (MCFA) and triacylglycerols emulsified by a sphingomyelin-rich phospholipid membrane (milk phospholipids, MPL). Infant formula comprises mainly long-chained fatty acids (LCFA) emulsified with dairy proteins and soy lecithin (SL) lacking sphingomyelin. Sphingomyelin content and saturation level of phospholipids affect the gut lipase activity, which alters the concentrations of lipid hydrolysis products in ileum and colon, and hereby putatively affects the competitive advantage of specific gut bacteria. Thus, differences in phospholipid and FA composition may modulate the establishment of the gut microbiota. We investigated effects of fatty acid (FA) composition and emulsification (MPL vs SL) ingested during establishment of human gut microbiota in germ-free mice, and found that cecal microbiotas from mice given MCFA-rich emulsions were characterized by high relative abundances of *Bacteroidaceae* and *Desulfovibrionaceae*, while LCFA-rich emulsions caused higher abundances of *Enterobacteriaceae*, *Erysipelotrichaceae*, *Coriobacteriaceae* and *Enterococcaceae*. Consumption of SL-emulsified lipids skewed the community towards more *Enterococcaceae* and *Enterobacteriaceae*, while MPL increased *Bacteroidaceae*, *Desulfovibrionaceae, Rikkenellaceae* and *Porphyromonadaceae*. Intake of SL increased cecal concentrations of iso-valeric and iso-butyric acids. This suggests that fat-type and emulsifiers applied in infant formula may have distinct effects on the establishment of the gut microbiota in formula-fed infants.

## Introduction

The composition of the gut microbiota is an important factor in human health, and has been suggested to affect host metabolism and immune homeostasis^1, 2^.

During birth and the first days of life, the gut microbiota starts to establish, and gradually develops into a complex microbial ecosystem^3^. The first colonizers are likely to play an important role, as the initial colonization may affect the composition of the gut microbiota throughout life.

The intestinal microbiota is highly dynamic during early life, and its development depends on many different factors including diet, environment and host metabolic processes^4–6^. Particularly, the very early diet is crucial for the colonization, as several studies have demonstrated a difference in composition of gut microbiota in formula-fed infants as compared to breast-fed infants^5, 7, 8^.

In human milk, lipids in the form of triacylglycerols (TAGs) constitute the principal energy source^9^. Although the fatty acid (FA) composition of the milk lipids to some extent reflects the maternal diet, the major FAs are always capric acid (C10:0), lauric acid (C12:0), myristic acid (C14:0), palmitic acid (C16:0), palmitoleic acid (C16:1), stearic acid (C18:0), oleic acid (C18:1) and linoleic acid (C18:2)^10–12^. The high content of medium-chain fatty acids (MCFAs) (C8:0-C12:0) is unique for milk. Human milk also contains low concentrations of a broad range of long-chain polyunsaturated fatty acids (LC-PUFAs)^13^. In infant formula the lipids are typically added in the form of vegetable oils^14^, and thereby comprise primarily C16 and C18 fatty acids (LCFAs), although long-chained polyunsaturated fatty acids (arachidonic, eicosapentaenoic and docosahexaenoic acid) are often also added in low concentrations.

In milk, lipids exist as milk fat globules (MFG) dispersed in the aqueous phase^15^. The core of the MFG contains the TAG, and the entire MFG is enveloped by a milk fat globule membrane (MFGM) consisting of phospholipids (PLs), glycolipids, proteins and cholesterol. This MFGM acts as emulsifier, and stabilizes the lipid droplets in the emulsion. The composition of the PL-fraction varies during lactation, and different studies report slightly different compositions, however the major PLs in human MFGM are sphingomyelin (SM, 30–45%), phosphatidylcholine (PC, 20–40%), phosphatidylethanolamine (PE, 15–25%), phosphatidylserine (PS, 10–20%) and phosphatidylinositol (PI, 5–10%)^16–18^. The lipid droplets in infant formulas are typically stabilized by a combination of dairy protein and a crude phospholipid fraction, lecithin, usually extracted from soy bean (soy lecithin, SL). The SL is dominated by PC but PE, PI and PS are also found^19^. The predominant difference between the PL content in human milk and infant formula is thus the high content of SM and more saturated PLs found in milk.

It has been suggested that the SM content of the lipid particle interface may influence the lipolytic rate in the intestine^20^. Additionally, our previous studies have shown that SM concentration and degree of saturation in the lipid droplet interface influence the *in vitro* hydrolytic activity of both gastric and pancreatic lipase^21^. Furthermore, breastfed infants have been reported to have higher gastric TAG hydrolysis than infants fed formula^22, 23^, which is also reflected in a higher fecal fat excretion in formula-fed infants than in breastfed^24^ and may partly be explained by differences in positional distribution of saturated FA in the TAGs present in breastmilk and formula^25^. This suggests that different amounts of lipid degradation products reach the lower, bacteria rich parts of the intestine in formula-fed infants as compared to breast-fed infants, which may be due to the different compositions of the lipid droplet interface. Consistently, it was recently shown that intake of certain emulsifiers *per se* can have detrimental impacts on the gut microbiota^26^.

Very little is known about the influence of dietary FA on the gut bacterial composition. It has been reported that some typical milk FAs, as well as sphingosine, a degradation product of SM, inhibits the growth of specific bacteria^27, 28^. In recent years, a few studies have demonstrated a correlation between dietary lipid-intake and composition of the gut microbiota. Zentek *et al*. showed that intake of encapsulated MCFAs leads to increased abundance of given *Lactobacillus* species in piglets^29^, and a study in broilers demonstrated how a MCFA-enriched diet prompted the growth of *Enterobacteriaceae* and some *Lactobacillus* species, while *Firmicutes* in general, hereunder several other *Lactobacillus* species as wells as the families *Micrococcaceae* and *Enterococcaceae* were suppressed^30^. In infants, the percentage of fat in the complementary diet has been reported to correlate negatively with gut microbial diversity^5^. Thus, variations in exposure of the lower gut to lipid metabolites may modify the microbial ecosystem during its establishment early in life.

MCFAs selectively influence the growth of different members of the infant gut microbiota *in vitro*, hereunder promote the growth of *Bifidobacteriacae* and lactobacilli^31^. We therefore hypothesized that dietary PL composition and MCFA modifies the composition of the gut microbiota during the early establishment phase. Both the PL-type used for emulsification and the FA composition of the TAG in infant formula -mimicking emulsions given during gut colonization might affect the microbial composition. We thus tested two hypotheses; i) intake of emulsion based on either SL or MPL and ii) levels of ingested MCFA differently affects the establishing microbial community.

## Materials and Methods

### Lipid emulsions

#### Emulsion preparation

Lipid emulsions were based on regular tap water with either RO (rapeseed oil, cold-pressed, organic) (Irma A/S, Albertslund, Denmark) or CO (coconut oil, cold-pressed, organic, virgin) (Irma A/S, Albertslund, Denmark). The RO represented LCFAs (C16–18), while the CO represented the MCFAs (C8-12), (Table 1). Lipids were emulsified in either SL (from soy beans, 90%, AppliChem, VWR, Darmstadt, Germany) or MPL purified from Lacprodan^®^ PL-20 (Arla, Viby J, Denmark) as detailed below. All emulsions contained thistle oil (cold-pressed, organic, filtered) (Urtekram Int. A/S, Mariager, Denmark) to supply essential fatty acids and increase fluidity of the CO. The total fat content was 4% comprising 3.4% w/w oil (78% RO or CO and 22% thistle oil) and 0.6% w/w emulsifier (SL or MPL).

**Table 1:** Fatty acid content in emulsions illustrated as percentage-wise distribution.

FA and PL	Fatty acid composition [%]		
	MPL/CO	MPL/RO	SL/RO
C6:0	0.5	0.0	0.0
C8:0	7.2	0.0	0.0
C10:0	5.9	0.0	0.0
C12:0	45.7	0.1	0.0
C14:0	17.5	0.7	0.1
C16:0	10.8	6.7	5.7
C16:1	0.1	0.3	0.2
C18:0	4.0	2.6	1.9
C18:1, n-9	3.2	60.0	57.8
C18:1, n-7	0.3	2.5	2.3
C18:2, n-6	4.3	18.0	23.3
C20:0	0.2	0.5	0.4
C18:3, n-3	0.1	7.7	7.2
C20:1, n-9	0.1	0.8	0.8
C22:0	0.1	0.2	0.3

The included lipid emulsions were RO emulsified in SL and MPL, respectively, as well as CO emulsified in MPL. The original protocol also contained the combination of CO and SL, but this did not fulfill our quality standards due to failure in maintenance of homogeneity. Emulsions were pre-homogenised using an Ultra-Turrax (Step 4) (Janke & Kunkel IKA-Labortechnik, Staufen, Germany) for 2 min followed by homogenization using a Rannie homogenisator (APV, Copenhagen, Denmark) at a pressure at 25 bar and 250 bar with four circulations. Emulsions were freshly prepared every other day. Emulsions containing RO were stored at +4 °C, and the emulsion containing CO was stored at room temperature.

#### Purification of milk phospholipids

MPL was purified from Lacprodan^®^ PL-20 (Arla, Viby J, Denmark) with a procedure modified from the European Patent Specification for *Method for extracting sphingomyelin*, published by H. Burling and L. Nyberg. Lipids were extracted from the milk protein concentrate using chloroform:methanol (1:1) for 1 hour with agitation. After centrifugation at 4000 g for 5 min, the supernatant was transferred to a new tube and organic solvents were evaporated on rotary evaporator. Any remaining lipids were re-extracted from the protein phase by repeating the extraction procedure. After evaporation, lipids were dissolved in *n*-heptane:Ethanol (1:2) in the ratio 1:3. The solution was separated by centrifugation at 4000 *g* for 5 min, and the *n*-heptane phase was transferred to a new tube and PLs were precipitated using ice-cold acetone in the ratio 1:1.5 (*n*-heptane:acetone) for 1 hour. After centrifugation, the supernatant was discarded and the PLs were washed in ice-cold *n*-heptane:acetone (1:1.4). The PLs were spun down and remaining organic solvents were evaporated using N_2_. Finally, PLs were freeze-dried overnight and stored at +20 °C until use.

#### Characterization of emulsifiers

SL and purified MPL were characterized by HPLC essentially as described by Silversand and Haux^32^, using a Luna HILIC column (100 × 3 mm) (Phenomenex) on an Agilent 1100 HPLC system with a Polymer Labs evaporative light scattering detector PL-ELS 2100 operated with an N_2_ flow of 1.90 ml/min, and an evaporator temperature of 90 °C and a nebulizer temperature of 75 °C. Calibration curves were run in parallel with sample analysis. Calibration curves included the PLs: lysoPC (C18:1), lysoPG (C18:1), PC (C18:1), PS (brain), PI (soy), PE (C18:1), PG (C18:1), glucosylceramide (C18:1), cardiolipin (bovine heart) and SM (18:1). To analyze non-polar contaminants, calibration curves containing cholesterol and TAG were also applied. All standard PLs were from Avanti Polar Lipids (Alabaster, AL, US). Calibration curves were made for all PL by injecting 0.25, 0.50, 1.0, 2.0, 4.0 and 6.0 µg of each lipid into the system. The PL purity of applied PLs was defined as the total PL mass- percentage of total dry weight.

Characterization of MPL revealed a PL purity of 87%, with traces of glycosylceramide, cholesterol and TAG constituting the remaining fraction. The PL composition was 26% PE, 26% PC, 29% SPH, 10% PI and 9% PS. Our characterization of the SL revealed a PC content of 95%, with only traces of lyso-PC and PI.

#### Characterization of fatty acids in emulsions

Lipids were extracted from emulsions using a modified version of the Bligh and Dyer extraction^33^. To a volume of 100 µl emulsion, 200 µg of TAG 19:0 was added as internal standard. Lipids were extracted in 500 µl methanol, 500 µl chloroform and 300 µl of a 0.73% NaCl-solution, followed by phase separation by centrifugation, and the lipid phase was transferred to a new vial and organic solvent was evaporated under N_2_. To ensure release of FAs also from the SM in MPL, lipids were hydrolysed according to Aveldano^34^, prior to methylation of the released FAs. The lipid hydrolysate was extracted in a volume of 2.0 ml chloroform followed by centrifugation for 5 min. Remaining lipids were re-extracted using another 1.0 ml chloroform. Lipid phases were compiled and chloroform was evaporated under N_2_. Released FAs were methylated and analyzed using GC-FID as earlier described^35^.

The FA composition in emulsions is shown in Table 1. As expected, the CO-containing emulsion was rich in C8:0-C12:0, while emulsions based on RO were rich in oleic (C18:1n-9) and linoleic (C18:2n-6) acid.

#### Stability of emulsions

To determine the stability of the emulsions over time, lipid oxidation and droplet size were measured on three successive days, indicated as Day 0, 1 and 2. Day 0 refers to the production day, Day 1 refers to the time point after 24 hrs at storage temperature and Day 2 refers to 24 hrs of storage followed by 24 hrs at mouse housing environment room temperature (RT). The emulsions measured at Day 2 were either stored at +4 °C for the first 24 hrs (emulsions containing RO) or at RT (emulsion containing CO), followed by 24 hrs at RT.

#### Lipid oxidation

Lipids were extracted from emulsions according to the method described by Bligh and Dyer^33^ with reduced amount of solvent applied^36^. The analysis was done in duplicate and further used for determination of peroxide value (PV). The PV was determined by a colorimetric method based on formation of an iron–thiocyanate complex measured according to the method described by Shantha and Decker^37^.

#### Droplet size

The size of the lipid droplets in the oil-in-water emulsion was determined by laser diffraction using a Mastersizer2000 coupled to a Hydro2000S (Malvern Instruments, Worcestershire, UK) according to manufacturer’s instruction. The emulsion was diluted directly in recirculating water (2000 rpm) reaching an obscuration of 12–15%. Refractive index was 1.445 for CO and 1.462 for RO.

In connection with oxidation status measured as peroxide value (PV, meq O2/kg oil), the emulsions stabilized by the highly saturated MPL, were highly stable, while the PV in the emulsion containing a relatively high concentration of PUFA (SL/RO) increased significantly over time. Regarding the particle size of lipid droplets, these were homogeneous and similar between all three emulsions over time ranging from 0.04–3 µm with a mean of around 0.5 µm, thus similar to lipid droplets in infant formulas^38^. Details from stability studies are found in Supplementary data (Figure S1 and S2).

### Preparation of inoculum based on infant feces

An inoculum was prepared by pooling of fecal samples obtained from nine healthy, breast-fed infants aged 2 to 5 months as previously described^31^. Anaerobic cultivation for two days on Brain-Heart Infusion plates revealed a bacterial concentration of at least 5.0 × 10^5^ CFU/100 µl. The inoculum was kept under anaerobic conditions until use. Informed consent for the use of the children’s samples in the study was obtained from all mothers, who had volunteered to donate an infant fecal sample but were not participating in a clinical trial.

### Animals and housing

The animal experiment was carried out at the DTU National Food Institute (Mørkhøj, Denmark) facilities. Ethical approval was given by the Danish Animal Experiments Inspectorate. The authorization number given is 2012-15-2934-00089 C2. The experiment was overseen by the National Food Institutes in-house Animal Welfare Committee for animal care and use and carried out in agreement with the guidelines of this institution.

Germ free Tac:SW outbred (Taconic Inc., NY, USA) male and female pups (n = 36) born by six dams were included in the study. Animals were kept in sterile isolators (Harlan Isotec and Bell or Scanbur A/S, Karlslunde, Denmark) until the onset of the study. The pups suckled by mothers fed with sterilized water and sterilized standard chow (Altromin, 1320 N, Brogaarden, Gentofte, Denmark). Germ-free conditions were continuously verified by aerobic and anaerobic cultivation of fecal samples.

After onset of the experiment (inoculation and diet as describes below), mice were housed individually in standard mouse cages (euro-standard type II, 267X207X140mm, Tecniplast, Varese, Italy) with access to bedding material, hiding-place and wooden block. The environment was maintained on a 12-h light/12-h dark cycle at a constant temperature (22 ± 1 °C) with air humidity at 55 ± 5% rel. humidity. Air was changed 8–10 times/hour in a room with excess pressure. Animals were overseen daily by licensed animal-technicians.

Throughout the study period, mice had *ad libitum* access to a very low-fat diet (880 mg crude fat/kg) (Altromin C1056, Brogaarden, Gentofte, Denmark). Drinking water was replaced with one of three types of lipid emulsions (details below). Emulsions were given *ad libitum*.

The study period was 14 days, where after the mice were euthanized in the animal facility by cervical dislocation as this is assumed to be ethically the most correct method. Mice were fasted for 4 hours prior to euthanasia.

### Diet and inoculation with infant feces suspension

At the age of 3 to 4 weeks, pups were transferred to a non-sterile environment and inoculated by gavage with 100 µl human infant feces diluted 1:1000 in pre-reduced PBS. Due to the non-fasted state of the animals and the relatively large volume of the inoculum, this unfortunately resulted in the need for immediate euthanisation of 6 out of the 36 animals, because a part of the inoculum could not be contained in the stomach and was obstructing the lungs. The remaining 30 pups appeared completely healthy and were distributed equally into three groups of 10 animals, randomized by litter and gender. One group, (MPL/CO) was fed with emulsions based on MPL and coconut oil (CO), another group, (MPL/RO) was fed with MPL and rapeseed oil (RO), while the third group (SL/RO) was fed with emulsion based on SL and RO.

## Sample preparation and analysis

### Sample collections

Fecal samples were collected from mouse cages at Day 2, 5 and 12. At the day of euthanasia, lumen contents from ileum and cecum were collected and stored at −80 °C until use. Throughout the study period, emulsion intake was measured every other day to give an average daily intake. Mice were weighed once a week.

### 16S rRNA Sequencing

#### Purification of bacterial DNA

From fecal samples and lumen content (ileum, cecum and colon), DNA was purified using QIAamp DNA stool minikit (Qiagen, Hilden, Germany) according to the manufacturer’s instructions. Prior to this, a bead beating step was included using Zirconia Silica Beads (Bio Spec Products Inc., Oklahoma, USA). DNA concentration was measured using Qubit® dsDNA HS Assay Kit (Invitrogen^TM^, Life Technologies, San Francisco, CA) with the Qubit® 2.0 Fluorometer. Purified DNA was stored at −20 °C until use.

#### Sequencing of 16S rRNA gene amplicons

Microbiota profiling was assessed by 16S rRNA gene sequencing on the Ion Torrent^TM^ platform as previously described^39^. Briefly, the V3-region of the 16S rRNA gene was amplified using a universal forward primer (PBU 5′-A-adapter-TCAG-barcode-CCTACGGGAGGCAGCAG-3′) with a unique 10–12 bp barcode for each bacterial community (IonXpress barcode as suggested by the supplier, Life Technologies) and a universal reverse primer (PBR 5′-trP1-adapter-ATTACCGCGGCTGCTGG-3′). Sequencing was carried out on an Ion OneTouch™ platform (Ion Torrent^TM^, Life Technology) using a 318-V2 chip. Sequence data were obtained in FASTQ format and further processed using CLC bio genomic workbench (Qiagen) in order to de-multiplex and remove sequencing primers and perform quality trimming. Sequence data are deposited in NCBI’s Sequence Read Archive (SRA) with the accession number SRP102024, BioProject number PRJNA376881.

#### Taxonomic assignment to 16S reads

Ribosomal Database Project Classifier software (RDP 10 database, Update 18) was used to classify the sequences^40^. Sequencing data were trimmed and processed using the CLC Genomic Workbench 7.0.3 (CLC Inc, Aarhus, Denmark) using the default parameters. Ribosomal Database Project (RDP) Classifier software (http://www.rdp.cme.msu.edu/) was used to classify the sequences using a 50% confidence threshold, maintained at the Ribosomal Database Project (RDP 10 database, Update 18).

### Assessment of Short Chain fatty Acids (SCFA) in cecal content

Quantification of SCFA in mouse cecal samples was carried out by Gas Chromatography as previously described^31^.

### Statistical analysis

Primary outcome was composition of the intestinal and fecal microbiota 14 days after inoculation, secondary outcome was effects on composition and concentration of short-chained fatty acids in cecum content feces.

Data are presented as Box & Whisker’s plot with 5–95% percentile. Statistical analysis was performed using the GraphPad Prism 5.00 software (GraphPad Software Inc., La Jolla, CA) and the R software package vers. 3.0.2 (www.r-project.org).

Two different nul-hypotheses were tested individually: 1) Intake of emulsions based on either SL or MPL during microbiota establishment lead to similar composition of the established microbiota; and 2) Increased intake of MCFA during microbiota establishment does not affect the established microbiota. For unpaired groups, differences were calculated using an unpaired t-test with Welch’s correction, and for paired groups, differences were calculated using a Wilcoxon matched pairs test. False discovery rates were calculated for all obtained p-values, and the adjusted p-values are stated in the following.

Differences were considered statistically significant if adjusted p-values < 0.05 were obtained. The distribution of each data set was tested using a D’Agostino & Pearson omnibus K2 normality test with a significance level at 0.05. Principal component analysis (PCA) was performed on auto-scaled data using the software-package LatentiX 2.11 (Latent5, www.latentix.com). Score-values for principal component (PC) 1, 2 and 3 were calculated for the three different groups (n = 10).

## Results

### Growth parameters and emulsion intake

Initial average weights (mean ± SD) per mouse were 17.5 ± 2.9 g for MPL/CO, 17.3 ± 5.3 g for MPL/RO and 17.4 ± 3.6 g for SL/RO. Throughout the study period, the total average weight gains per mouse were 10.1 ± 5.8 g for MPL/CO, 9.38 ± 3.7 g for MPL/RO and 10.5 ± 4.2 g for SL/RO. The average daily emulsion intake was 8.0 ± 2.0 ml for MPL/CO, 8.3 ± 4.2 ml for MPL/RO and 10.7 ± 2.9 ml for SL/RO. No significant differences were observed between groups for any of these parameters. Food intake was not measured.

### Colonization of the gut microbiota

#### Bacterial distribution in inoculum derived from infants

The bacterial distribution in inoculum derived from infant feces at phylum and family level is illustrated in Supplementary data (Figure S3).

At phylum level, the *Actinobacteria* comprised 91.5% of the total phyla, while *Firmicutes* constituted 7.0%. *Bacteroidetes* and *Proteobacteria* both accounted for approximately 0.5%, while less than 0.25% of the phyla were characterized as *unclassified*. At family level *Bifidobacteriaceae* and *Coriobacteriaceae* accounted for 72.8% and 18.3%, respectively. The family *Lachnospiraceae* accounted for 3.7%, while *Enterococcaceae* accounted for 0.9%. *Lactobacillaceae* and *Streptococcaceae* both accounted for approximately 0.5%. *Bacteroidaceae, Porphyromonadaceae, Rikenellaceae, Enterobacteriaceae, Sutterellaceae, Veillonellaceae, Acidaminococcaceae, Clostridiaceae, Peptostreptococcaceae, Ruminococcaceae and Erysipelotrichaceae* were all present; however, at levels below 0.5%. The remaining 3.6% of the families either belonged to families represented by less than 0.02% or by families that could not be classified by the RDB Classifier.

#### Colonized microbial communities at Day 2, 5 and 12

**Figure 1:**
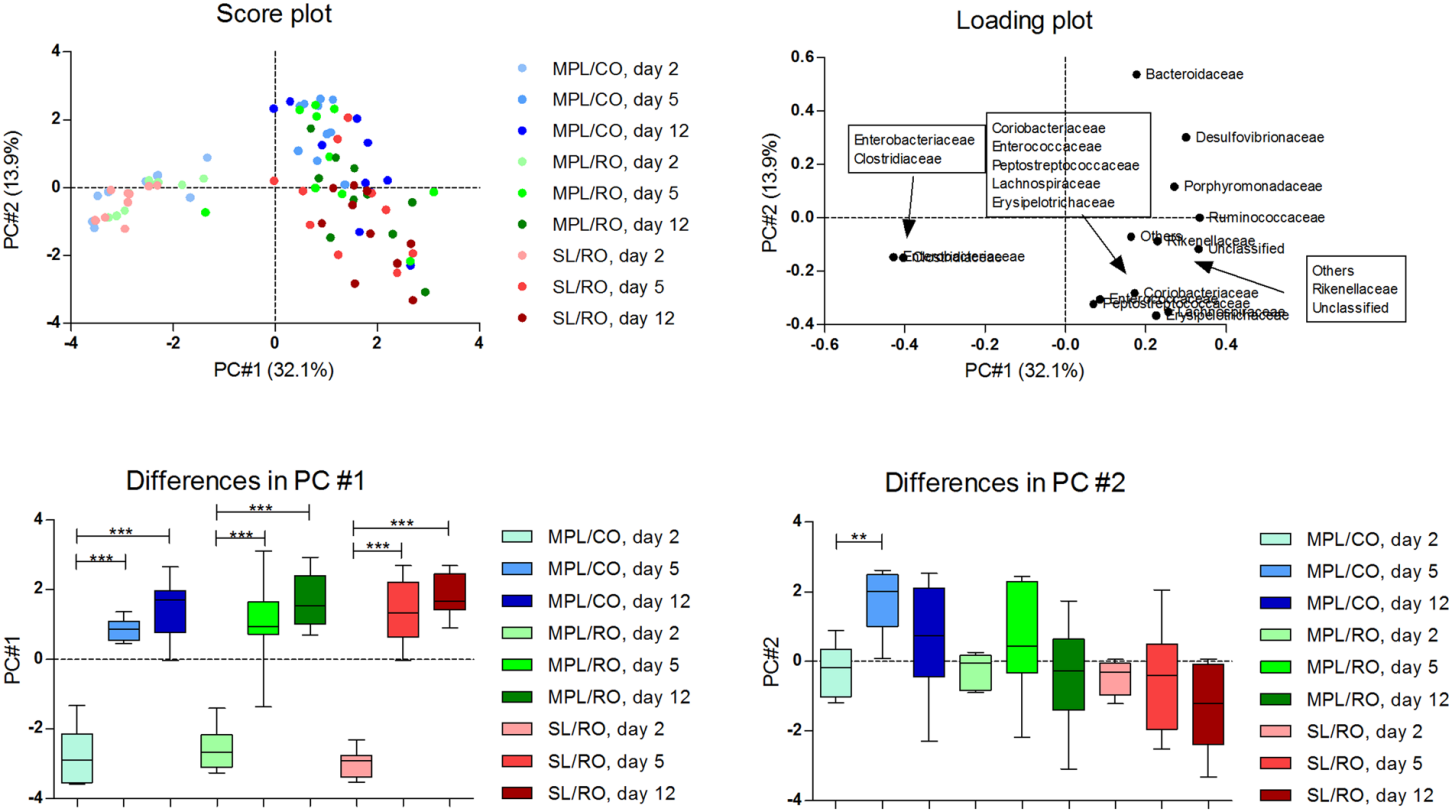
Fecal bacterial composition in mice at bacterial family level at Day 2, 5 and 12 after emulsion consumption, as assessed by Principal Component Analysis. (**A**) Score plot, (**B**) Loading plot, (**C** and **D**) Differences between days by PC #1 (**C**) and PC #2 (**D**) calculated by a repeated measures one-way ANOVA using a Tukey’s test as post-test. Differences between groups were calculated using an unpaired t-test with Welch correction. Colors refer to the three different lipid emulsions: MPL/CO (blue), MPL/RO (green) and SL/RO (red). Color intensity increases with number of days. On the loading plot (**B**), samples are distributed by the PC #1 (32.104%) and PC #2 (13.932%). Asterisks indicate significant differences (*p < 0.05, **p < 0.01, ***p < 0.001). PC: Principal Component, ANOVA: Analysis of variance, MPL, Milk phospholipids, CO: Coconut oil, RO, Rapeseed oil; SL: Soy lecithin.

Figure 1 illustrates the bacterial distribution at family level in fecal samples collected at Day 2, 5 and 12. In all groups, the bacterial composition at Day 2, characterized by *Enterobacteriaceae* and *Clostridiaceae*, deviated significantly from the other days at PC1 (p < 0.001) (Fig. 1 **A-C**). At Day 5 and 12 the bacterial distribution shifted towards higher PC1 values (PC1 ≥ 0), characterized by higher relative abundances of *Bacteroidaceae, Desulfovibrionaceae, Porphyromonadaceae, Ruminococcaceae, Rikenellaceae, Coriobacteriaceae, Enterococcaceae, Peptostreptococcaceae* and *Erysipelotrichaceae* (Fig. 1B). No differences were observed between Day 5 and 12.

**Figure 2:**
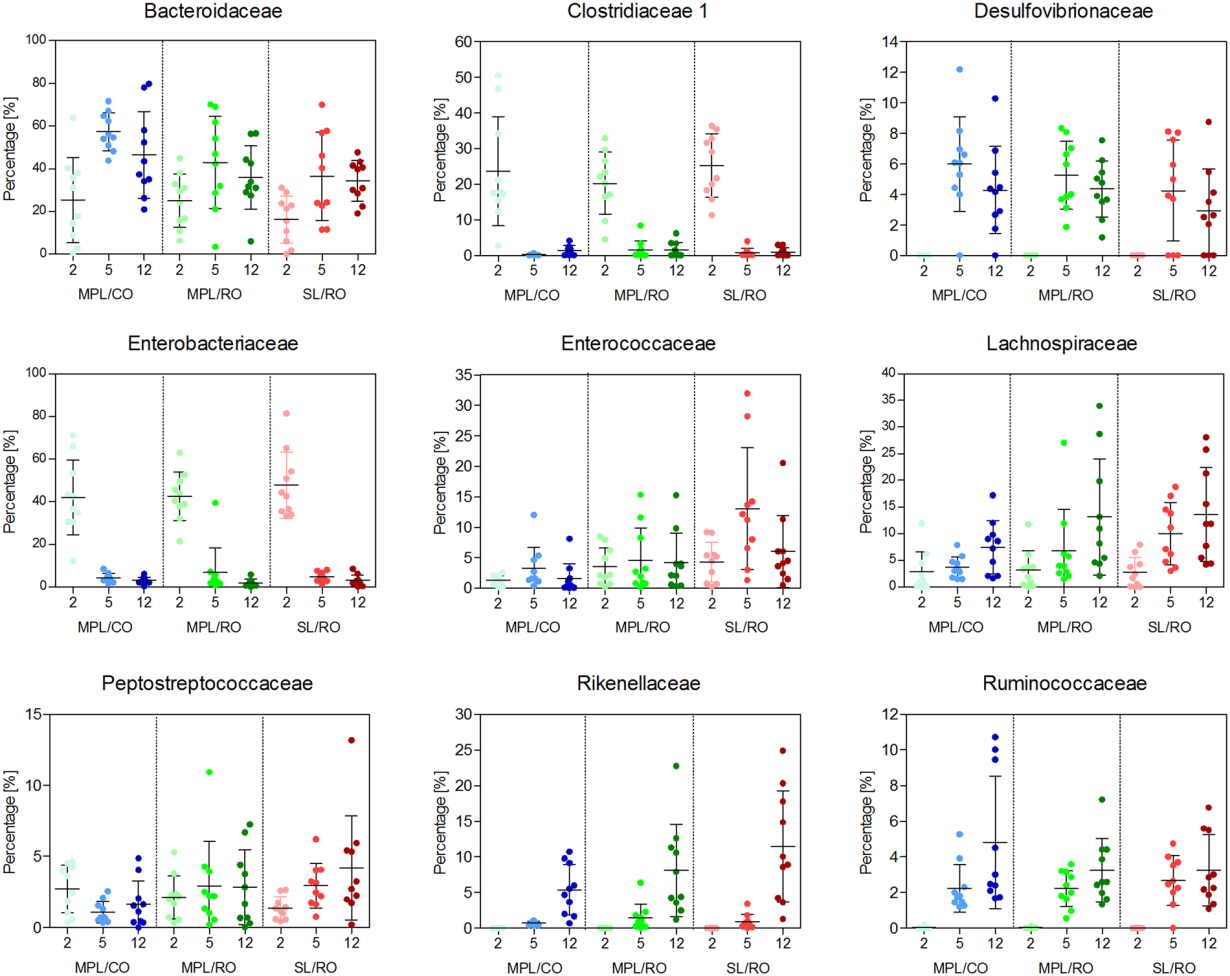
Percentage-wise distribution of bacterial families in fecal samples of mice fed with emulsions. Distribution at bacterial family level in feces samples from Days 2, 5 and 12. Distributions are shown for (**A**) *Bacteroidaceae*, (**B**) *Clostridiaceae* 1, (**C**) *Desulfovibrionaceae*, (**D**) *Enterobacteriaceae*, (**E**) *Enterococcaceae*, (**F**) *Lachnospiraceae*, (**G**) *Peptostreptococcaecae*, H: *Rikenellaceae* and I: *Ruminococcaceae*. Data are shown as mean percentages (*n* = 10). Error bars designate standard deviations. No differences were observed between groups within the same day. Note that Y-axis ranges are not identical. MPL: Milk phospholipids, CO: Coconut oil, RO: Rapeseed oil, SL: Soy lecithin.

Figure 2 illustrates percentage-wise bacterial composition in fecal samples from Day 2, 5 and 12. Levels of *Bacteroidaceae*, *Desulfovibrionaceae*, *Lachnospiraceae*, and *Ruminococcaceae* increased from Day 2 to Day 5. *Lachnospiraceae* increased steadily during the period, while other families increased notably from either a relatively high level (*Bacteroidaceae*) or an undetectable level (*Desulfovibrionaceae* and *Ruminococcaceae*). Levels of *Enterococcaceae* and *Peptostreptococcaceae* remained stable over the period.

For *Clostridiaceae 1*and *Enterobacteriaceae*, there was a high level at Day 2, whereafter these families disappeared again. Both of these families constituted less than 0.5% in inoculum, but at Day2, represented 20–25% and 40% of the colonized bacterial communities, respectively. Interestingly, *Rikenellaceae* increased markedly between Day 5 and Day 12, but constituted undetectable or very low levels at Days 2 and 5. Despite the fact that *Bifidobacteriaceae* were dominating the inoculum, these were completely absent from feces already at Day 2.

#### Bacterial distribution in lumen

**Figure 3:**
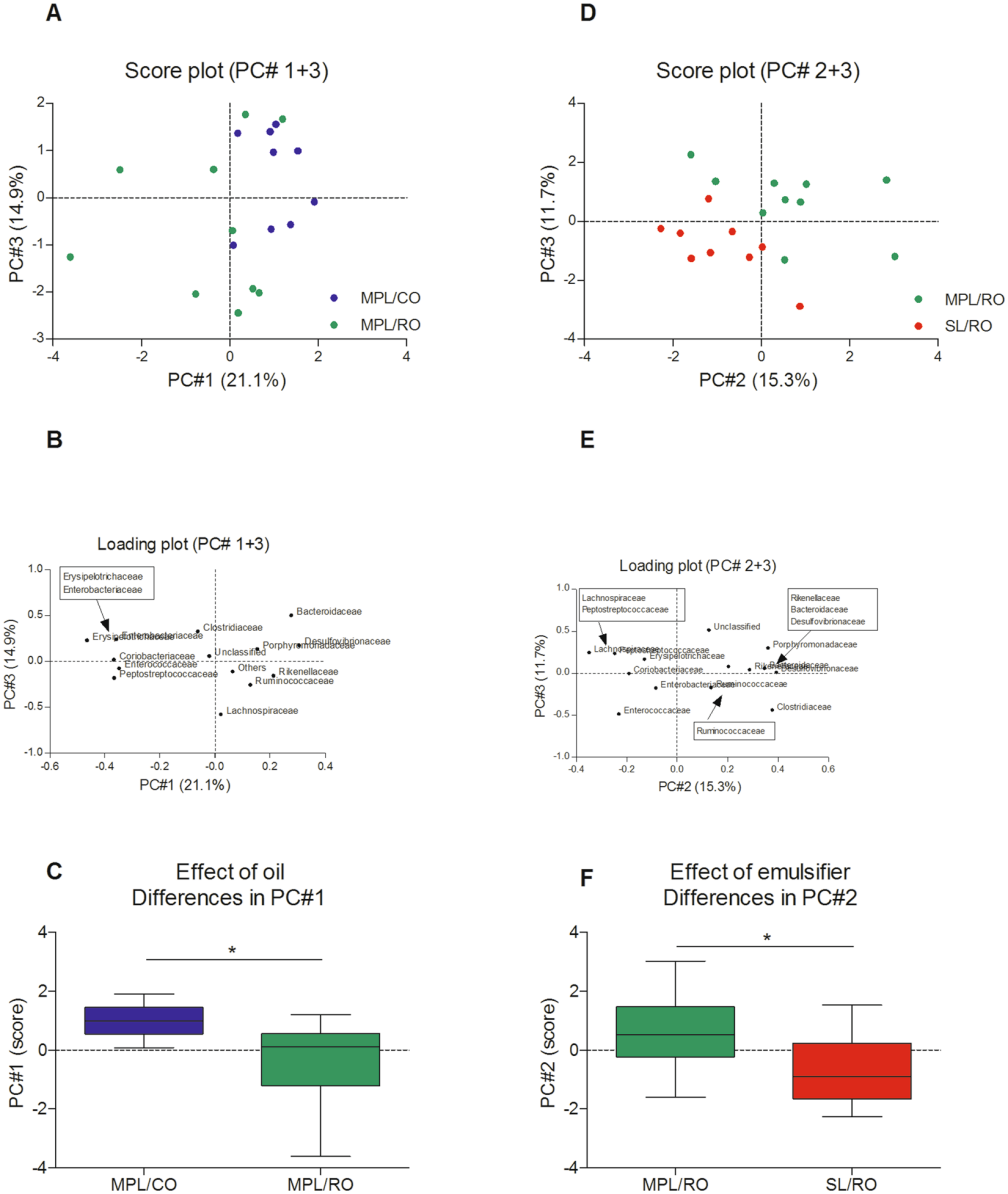
Effect of oil (left) and type of emulsifier (right) on the bacterial family composition in cecum as assessed by Principal Component Analysis. Score-plots (**A** and **D**), Loading plots (**B** and **E**), and differences in PC1 for the oil effect (**C**) and PC2 for the emulsifier effect (**F**) calculated using an unpaired t-test with Welch’s correction. Asterisks indicate significant differences (*p < 0.05). PC: Principal Component, MPL: Milk phospholipids, CO: Coconut oil, RO, Rapeseed oil, SL: Soy lecithin.

***Ileum, cecum and colon*** **:** Type of emulsifier and oil, respectively, were tested for effect on bacterial composition in ileum, cecum and colon. No effect was observed on bacterial composition in ileum and colon (Supplementary data, Figure S4), while the microbiota in cecum was significantly influenced. Both type of oil and type of emulsifier affected the composition of the cecal microbiota (Fig. 3).

**Figure 4:**
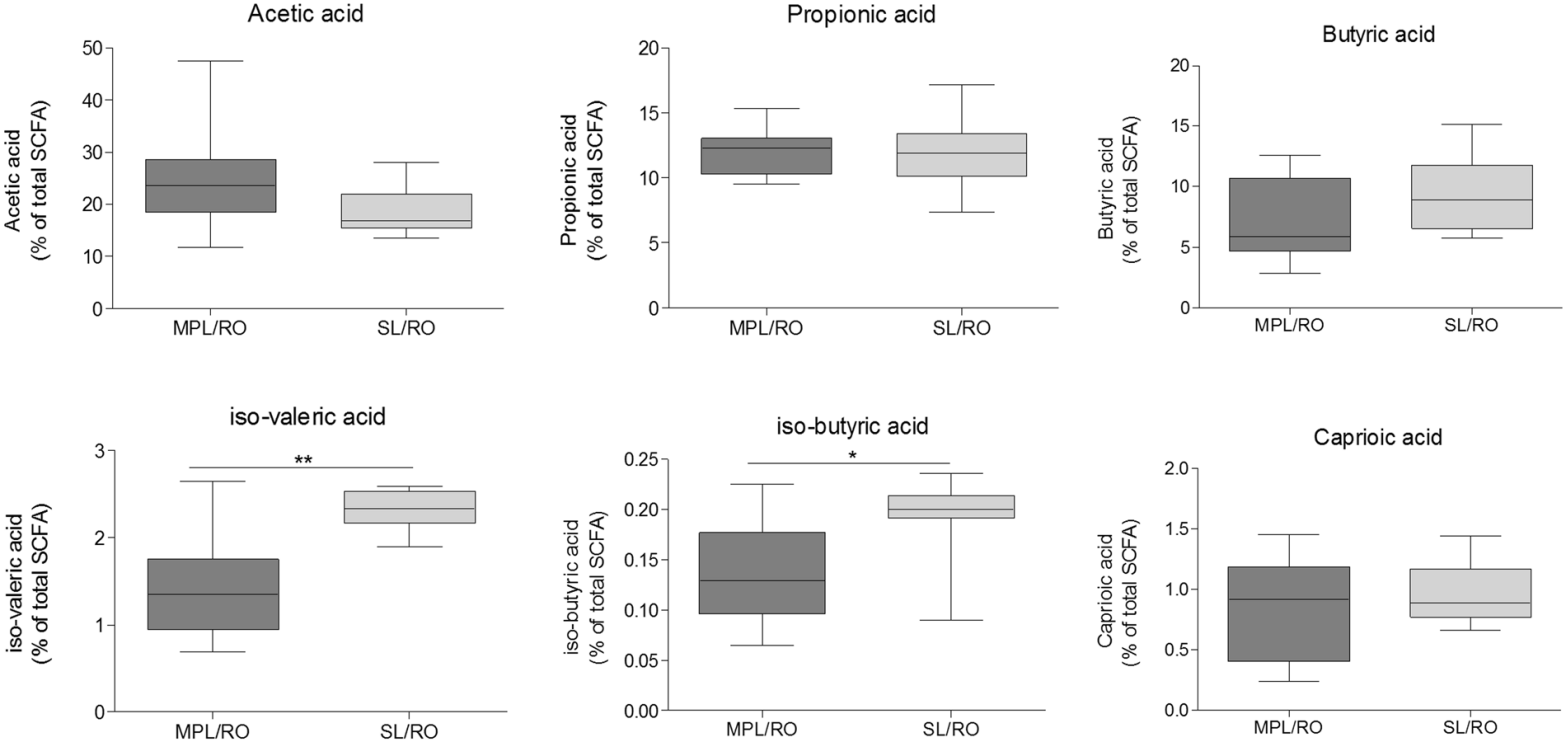
Effect of emulsifier on short-chain fatty acid (SCFA) production in cecum of mice at Day 14 after emulsion consumption. Levels of SCFAs are illustrated as percentages of total SCFAs. (**A**) Acetic acid, (**B**) Propionic acid, (**C**) Butyric acid, (**D**) iso-valeric acid, (**E**) iso-butyric acid, and (**F**) Caprioic acid. Differences between groups were calculated using unpaired t-test with Welch correction. Asterisks indicate significant differences (*p < 0.05, **p < 0.01). MPL: Milk phospholipids, RO, Rapeseed oil, SL: Soy lecithin.

Separation along PC1 and PC3 reveals that MPL/CO samples, clustering at PC1 > 0 (Fig. 3A), and characterized by higher relative abundances of *Bacteroidaceae*, *Desulfovibrionaceae*, *Porphyromonadaceae*, *Rikenellaceae* and *Ruminococcaceae* (Fig. 3B), were different from MPL/RO samples, clustering at PC ≤ 0 and characterized by *Enterobacteriaceae*, *Erysipelotrichaceae*, *Coriobacteriaceae*, *Enterococcaceae*, and *Peptostreptococcaceae*. Scores vary more along both PC1 and PC3 for MPL/RO samples than for MPL/CO samples. Statistical analysis of differences in PC1 revealed that the separation between groups based on the intake of CO vs RO was significant (p = 0.02) (Fig. 3C). Assessment of the effect of the PL emulsifier showed that the SL/RO samples clustered at PC1and PC3 < 0 and were characterized by higher relative abundances of *Enterobacteriaceae* and *Enterococcaceae*, while MPL/RO samples clustered primarily at PC2 and PC3 > 0, and were primarily characterized by the relatively lower concentration of these families and a higher relative abundance of particularly *Porphyromodaceae* (Figs 3D and 4E). Statistical analysis of PC2 values revealed a significant difference in cecal microbiota composition between animals fed with MPL and SL, respectively (p = 0.04) (Fig. 3F).

At the individual bacterial family level, only a few families were significantly influenced by type of oil (Supplementary data, Figure S5). Intake of CO emulsified in MPL significantly increased the relative abundance of *Bacteroidaceae (*p = 0.033), but decreased the relative abundance of *Coriobacteriaceae* (p = 0.019), as compared to the RO group.

#### Short-chain fatty acids (SCFA) in cecum

While there were no difference in cecal concentrations of SCFA between the groups of mice fed the different oil types (Supplementary data, Figure S6), comparison of mice fed with oils emulsified with SL and MPL, respectively, revealed significant differences in the cecal concentrations of branched SCFA, but not total SCFA content. Total cecal SCFA contents from the animals fed MPL/RO and SL/RO were 26.6 ± 6.8 nmol/mg and 25.0 ± 5.0 nmol/mg, respectively. Acetic acid constituted 20–25% of the total SCFA in cecum (Fig. 4A), propionic comprised approximately 12% of total SCFA in both groups (Fig. 4B) while butyric acid constituted 7–10% of the total SCFAs (Fig. 4C). No differences were observed between the SL/RO and MPL/RO groups for these SCFA. However, the percentages of iso-valeric and iso-butyric acid were significantly (p = 0.001) higher in SL/RO than in MPL/RO (Fig. 4D and E). Caprioic acid constituted approximately 1% of the total SCFAs in both groups (Fig. 4F).

## Discussion

This study aimed to investigate whether intake of oil types characterized by either MCFA or LCFA, and of emulsions based on SL or MPLs during infant microbiota establishment led to differences in the bacterial composition. Germ-free mice inoculated with infant microbiota were applied as a model of the infant intestine, as germ-free mice colonized with human microbiota constitute a well-established tool for studies of ecology and metabolism of intestinal bacteria, as well as of their interaction with dietary components^41–43^. We thus aimed to mimic the influence of diet during the colonization phase of the newborn (germ-free) intestinal environment. As the immune system is under rapid development after birth and during childhood, we chose to inoculate the mouse pups with human bacteria as early as possible, i.e. right after weaning, at age 3–4 weeks. It should however be noted, that this exposure to a complex microbiota occurred later than it would be the case in nature. Furthermore, it has recently been established that the human gut is probably not completely germ-free at birth^44^. The animal model applied here was thus not designed to mimic effects of the establishing microbiota on the host health and immune system, but was specifically intended to elucidate the effect of dietary lipids on an establishing human gut microbiota in an intestinal environment.

16S rRNA sequencing revealed that both type of oil and type of emulsifier, when consumed as emulsions in drinking water, significantly influenced the composition of the cecal gut microbiota in originally germ-free mice inoculated with human infant microbiota. Intake of CO, rich in MCFAs, lead to a microbiota characterized by higher relative levels of *Bacteroidaceae*, *Desulfovibrionaceae*, *Porphyromonadaceae*, *Rikenellaceae* and *Ruminococcaceae*, while intake of RO, rich in C18 unsaturated FAs, lead to a microbiota characterized by higher relative abundances of *Enterobacteriaceae*, *Erysipelotrichaceae*, *Coriobacteriaceae*, *Enterococcaceae*, and *Peptostreptococcaceae* (Fig. 3). Several studies have revealed that MCFAs, especially C10:0 and C12:0 FAs, function as antibacterial agents against specific bacteria *in vitro* ^28, 45^. Additionally, *in vivo* studies have demonstrated that a high intake of MCFAs suppresses the growth of *Enterococcaceae* in broiler chickens^30^, which is in accordance with the observations reported here. However, a high intake of MCFA has also been reported to promote the growth of *Enterobacteriaceae* in chickens and piglets^29, 30^, while the opposite was observed in present study. Several *in vitro* studies have demonstrated that certain C18-FAs are capable of improving the growth of *Lactobacillus* ^46, 47^, while we recently showed that MCFA given as free FAs and monoacylglycerol promoted growth of both *Lactobacillus* and *Bifidobacterium* during *in vitro* fermentation by human infant fecal microbiota^31^. Hence, the differences in gut microbiota establishment caused by intake of the different types of oil may be a consequence of specific antibacterial activities as well as growth promoting properties of the FAs.

Univariate statistical analysis showed that only a few bacterial families were significantly differently affected by the type of emulsifier, but when considering the entire community composition by PCA, significant effects were observed.

Emulsification in SL thus led to a bacterial composition characterized by more *Enterobacteriaceae* and *Enterococcaceae*, while emulsification in MPL led to a relative increase of *Porphyromonadaseae* (Fig. 3D and E). As it is known that the presence of SM in emulsifiers reduce the hydrolytic activity of the co-lipase/pancreatic lipase complex^20, 21^, it is likely that the content of SM in the MPL-stabilized emulsion influenced the lipase activity in the gut of mice fed with this type of emulsifier. We have previously demonstrated slower lipid absorption from MPL-stabilized emulsions than from SL-stabilized, putatively leading to a higher concentration of fatty acids in the distal gut^21^. Although this difference in exposure of the microbiota to fat might directly affect the growth of specific bacterial groups and thereby cause the observed differences, we cannot exclude other mechanism of action, e. g. increased retention-time of unhydrolyzed fat in duodenum causes enhanced bile-secretion, which may have substantial impact on the community composition.

A functional effect of altered gut microbiota in cecum may be reflected by altered levels of SCFAs. However, the differences in composition of gut microbiota obtained by intake of either MCFAs (high *Bacteroidaceae* and *Coriobacteriaceae*) or LCFAs were not reflected by different levels of SCFA in cecum (Figure S6). The levels of acetic, butyric, propionic and caprioic acids were similar in all three groups and were thus not affected by oil type neither by type of emulsifier. However, the levels of isovaleric and isobutyric acids were significantly higher in ceca of mice fed SL-stabilized emulsions than in mice fed MPL-stabilized emulsions. While most SCFAs are derived from fermentation of carbohydrates, iso-butyric and isovaleric acid typically derive from bacterial metabolism of proteins^48^, particularly leucine^49^. Concentrations of these two branched-chain amino acids are typically strongly correlated to each other^50^, reflecting an even influx of endogenous protein to the large intestine, possibly from the large source of endogenous protein originating from sloughed intestinal cells. The microbiota established during intake of the SL-stabilized emulsion was characterized by a higher relative content of Enterococcaceae (Fig. 3E). We speculate that this may have contributed to the proteolytic activity of the bacterial community, and thus to an increased production of iso-valeric and iso-butyric acids. Alternatively the SL-stabilized emulsion may have influenced the intestinal cell turnover, thereby liberating more protein to be digested by the gut bacteria.

In spite of the high numbers of *Actinobacteria* (91.5%), comprising *Bifidobacteriaceae* (72.8%) and *Coriobacteriaceae* (18.3%), present in the inoculum derived from infant feces (Supplemental Figure 3 **)**, these bacteria were not established in the mouse gut. After 14 days the amount of *Bifidobacteriaceae* was below 0.02% in ileum, cecum and colon of most mice, while *Coriobacteriaceae* were present at levels below 0.5%. The *Bifidobacteriaceae* were undetectable in fecal samples obtained at Days 2, 5 and 12, while *Coriobacteriaceae* emerged at very low abundance from Day 5. A recent study from our lab has revealed that approximately 60% of the bacterial genera present in samples from human adolescents are capable of establishment in the gut of germ-free mice^43^. However, it is well described that bifidobacteria are typically not as abundant in mice as in humans, although their prevalence depend highly on animal provider and batch^51^. We speculate that in the current model, the major reason for the lacking ability of the bifidobacteria to establish was likely to be that human breast milk contains oligosaccharides that are highly selective for the bifidobacterial genera typically present in breastfed infants^52^, while this selective factor was absent in the gut of the pups. However, we find that the lack of bifidobacteria did not necessarily affect the relevance of the mouse model for studies of effects of dietary factors on other bacterial strains.

Within each group, the bacterial composition was seen to be quite similar at Days 5 and 12, while a different microbiota was present at Day 2 (Fig. 1). This suggests that the major bacterial colonization took place between Day 2 and Day 5, and that thereafter the gut microbiota remained more or less unchanged. This emphasizes the importance of the very early environmental factors influencing bacterial establishment. The rapid and large changes occurring were demonstrated by the fact that *Clostridiacaeae* and *Enterobacteriaceae* constituted 20–25% and 40% of the fecal bacteria, respectively, at Day 2, but disappeared again at Day 5 (Fig. 2). For the *Enterobacteriaceaea*, this may be explained by their facultative nature, which may have given them a competitive advantage at the oxygen levels present in the germ-free gut, while they were outcompeted when the oxygen was later depleted by the colonized microbiota.

On a general level, the colonization pattern illustrated here is in accordance with the development of the fecal flora of newborn infants previously described^3, 53^, suggesting that the germ-free mouse gut, exposed to human infant microbiota, is applicable as a model for colonization of the human infant gut, although the lacking ability of the human-derived *Bifidobacteriaceae* to colonize the mice is important to consider.

## Conclusion

While e.g. the effect of breastfeeding and dietary fibres on the infant microbiota has been extensively studied^52^, studies of effects of lipids on gut microbes are scarce. Our results demonstrate that the type of emulsifier as well as the type of oil consumed during intestinal colonization influences the resulting microbial community structure 14 days after inoculation. It was particularly noteworthy that higher cecal concentrations of branched SCFA were observed in animals fed fed with SL-based emulsion, suggesting that SL emulsification leads to a higher degree of microbial protein degradation, than MPL emulsified fat. Our results indicates that the type of oil and emulsifier applied in infant formula affects establishment of the gut microbiota in infants, and may also influence the overall metabolic activity of the establishing bacterial community.

## Electronic supplementary material

Supplmentary Figures
