# Supplementary material for: Fatty acid composition and phospholipid types used in infant formulas modifies the establishment of human gut bacteria in germ-free mice
Source: Sci Rep. 2017 Jun 21;7:3975. doi: 10.1038/s41598-017-04298-0 (PMC5479857; doi:10.1038/s41598-017-04298-0)
Supplement: Supplementary file 1 — Supplmentary Figures [file 41598_2017_4298_MOESM1_ESM.pdf]

Supplementary data for:

Fatty acid composition and phospholipid types used in infant formulas modifies the establishment of human gut bacteria in germ-free mice

by

Rikke Guldhammer Nejrup, Tine Rask Licht and Lars Ingvar Hellgren

Content:

Supplementary Figures S1-S6.

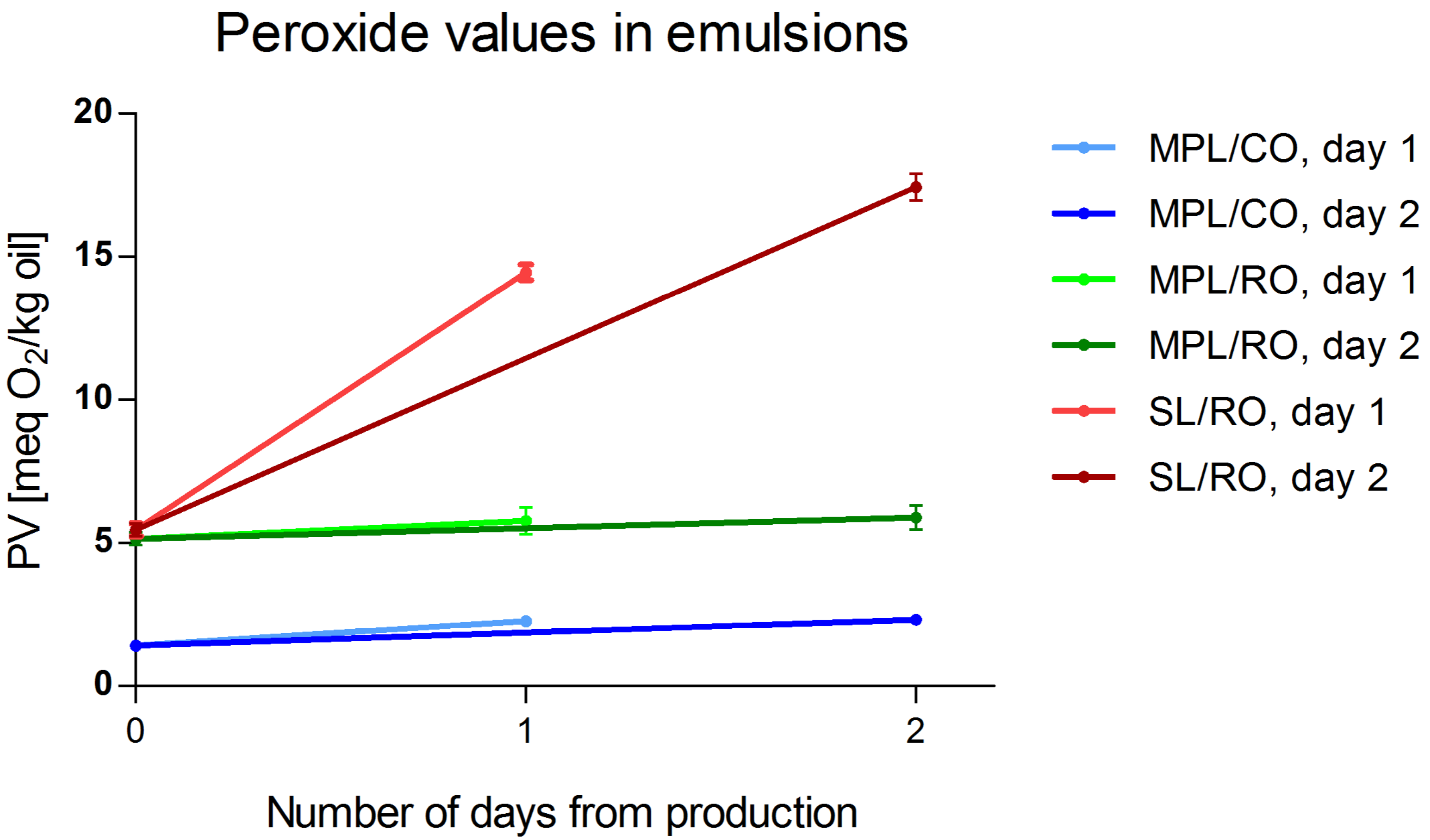

Figure S1 Peroxide values in emulsions over time. Values are shown in milliequivalent (meq) O2/kg oil. Values are measured right after production (day 0), and here after at day 1 and 2, respectively. Day 1 refers to the measurement after 24 hrs at RT, while day 2 refers to 24 hrs storage (+4℃ for MPL/Rapeseed (RO) and SL/RO, RT for MPL/Coconut oil (CO)) followed by 24 hrs at RT

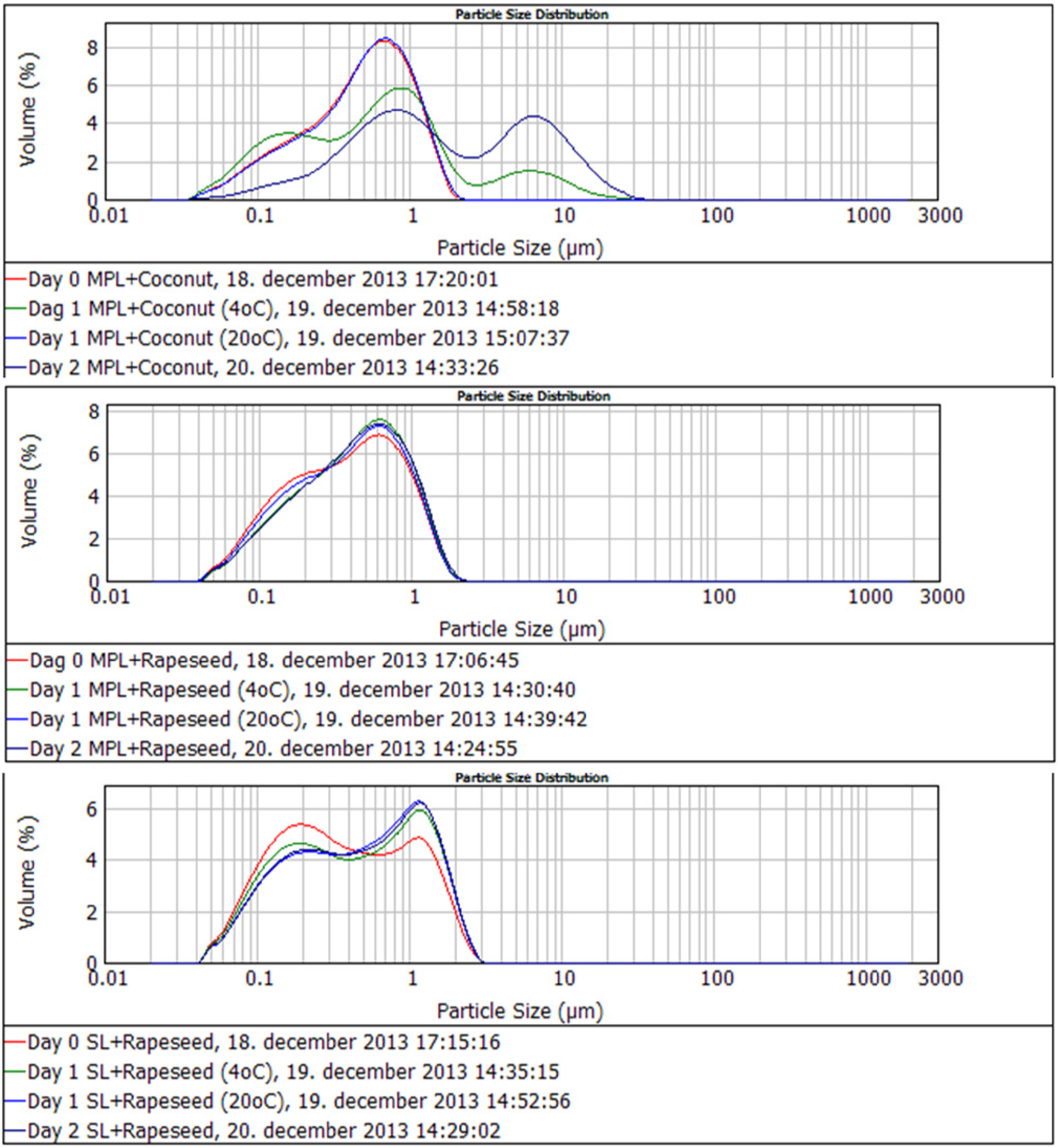

Figure S2 Particle size [µm] distribution of lipid droplets in emulsions. A: MPL/CO, B: MPL/RO, C: SL/RO. Colors refer to: day 0 (red) corresponding to the production day, day 1 after storage at +4°C for 24 hrs (green), day 1 after 24 hrs at +20°C (light blue) and day 2 after storage at +4oC for 24 hrs followed by 24 hrs at +20°C (dark blue).

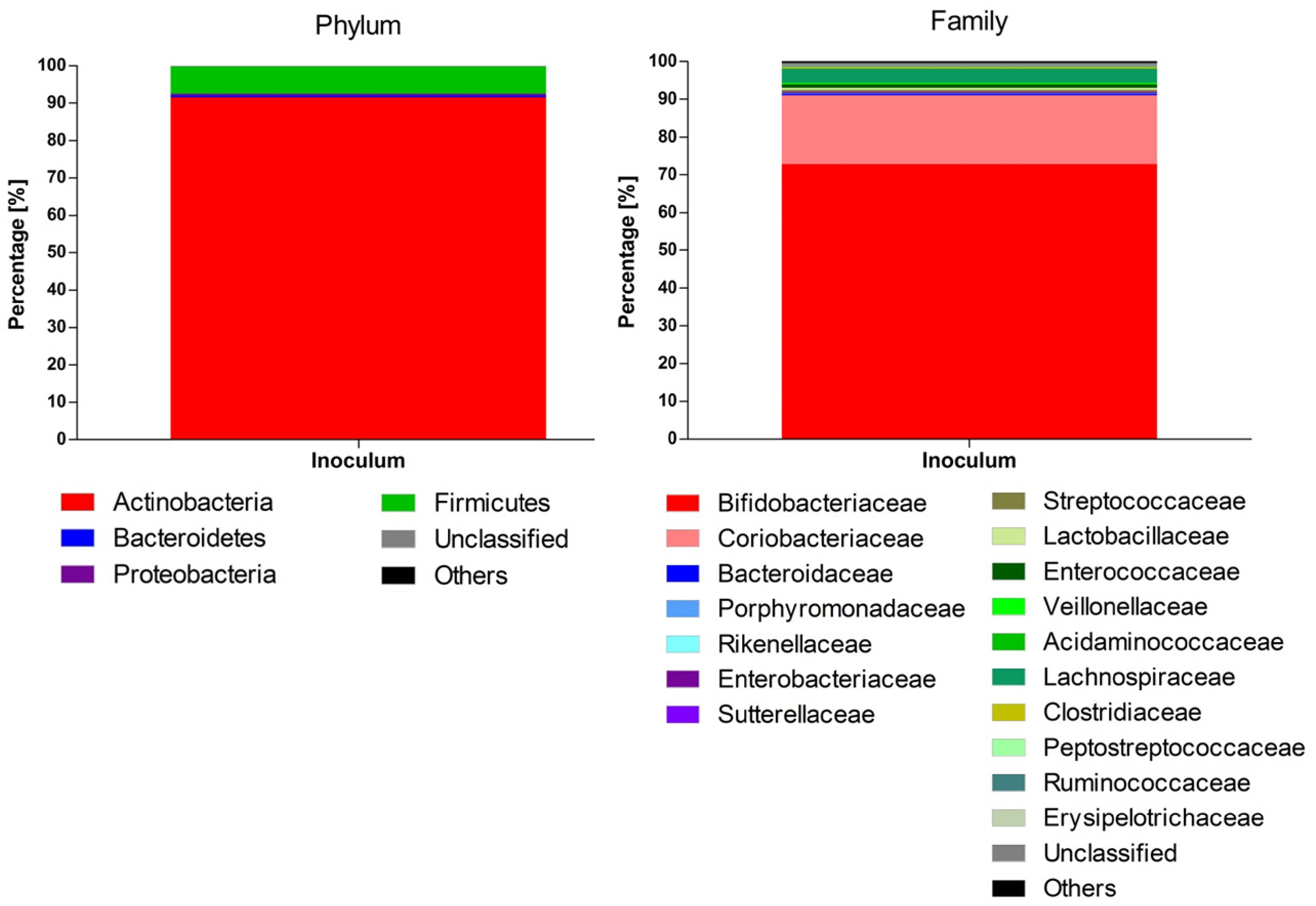

Figure S3 Bacterial distribution in inoculum. Left: phylum level, Right: Family level. Color matches between families and their corresponding phylum.

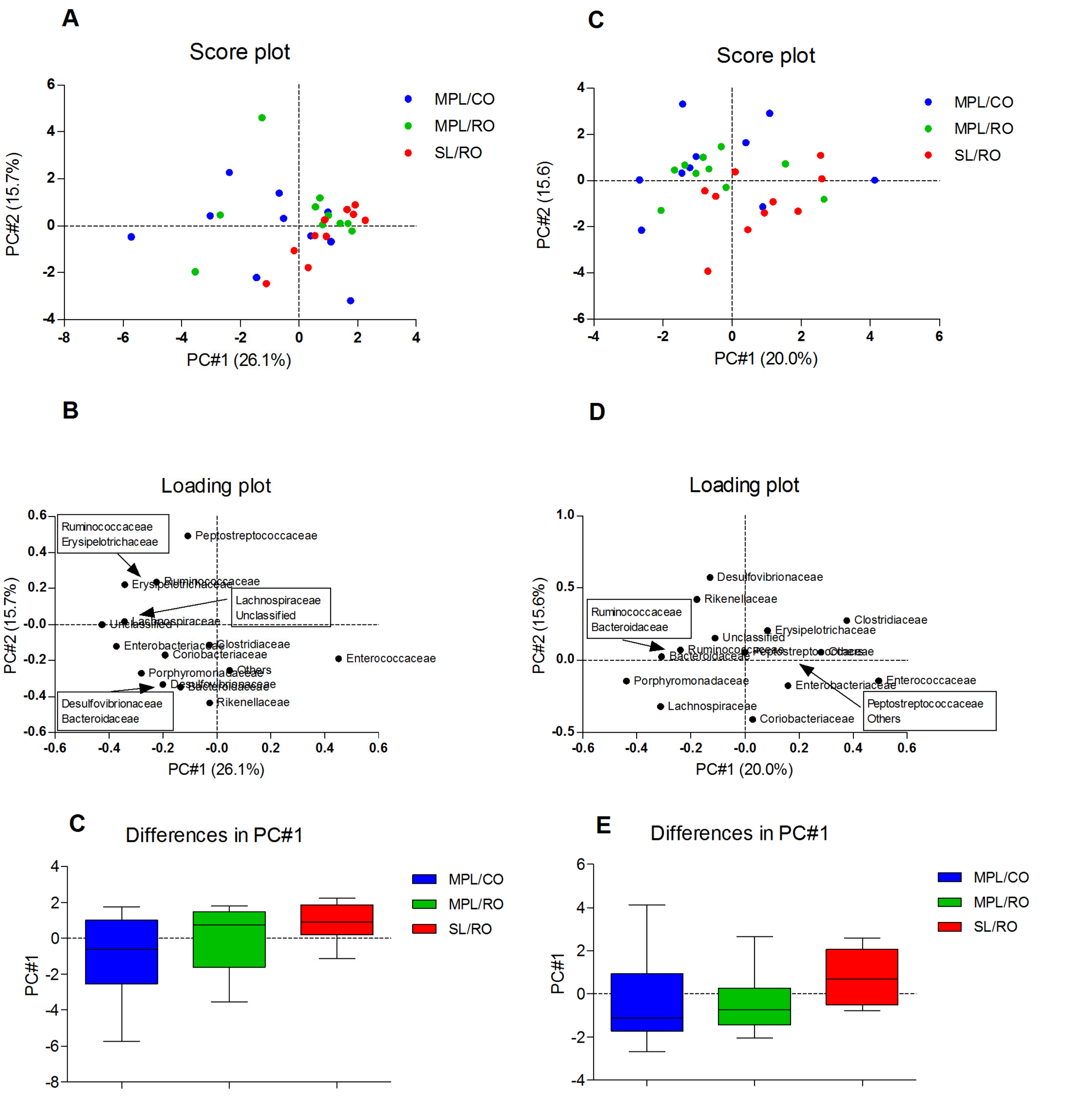

Figure S4 Effect of oil and type of emulsifier on the bacterial composition in ileum (left) and colon (right) illustrated in a PCA. Differences between groups are calculated using an unpaired t-test with Welch's correction on PC1 values.

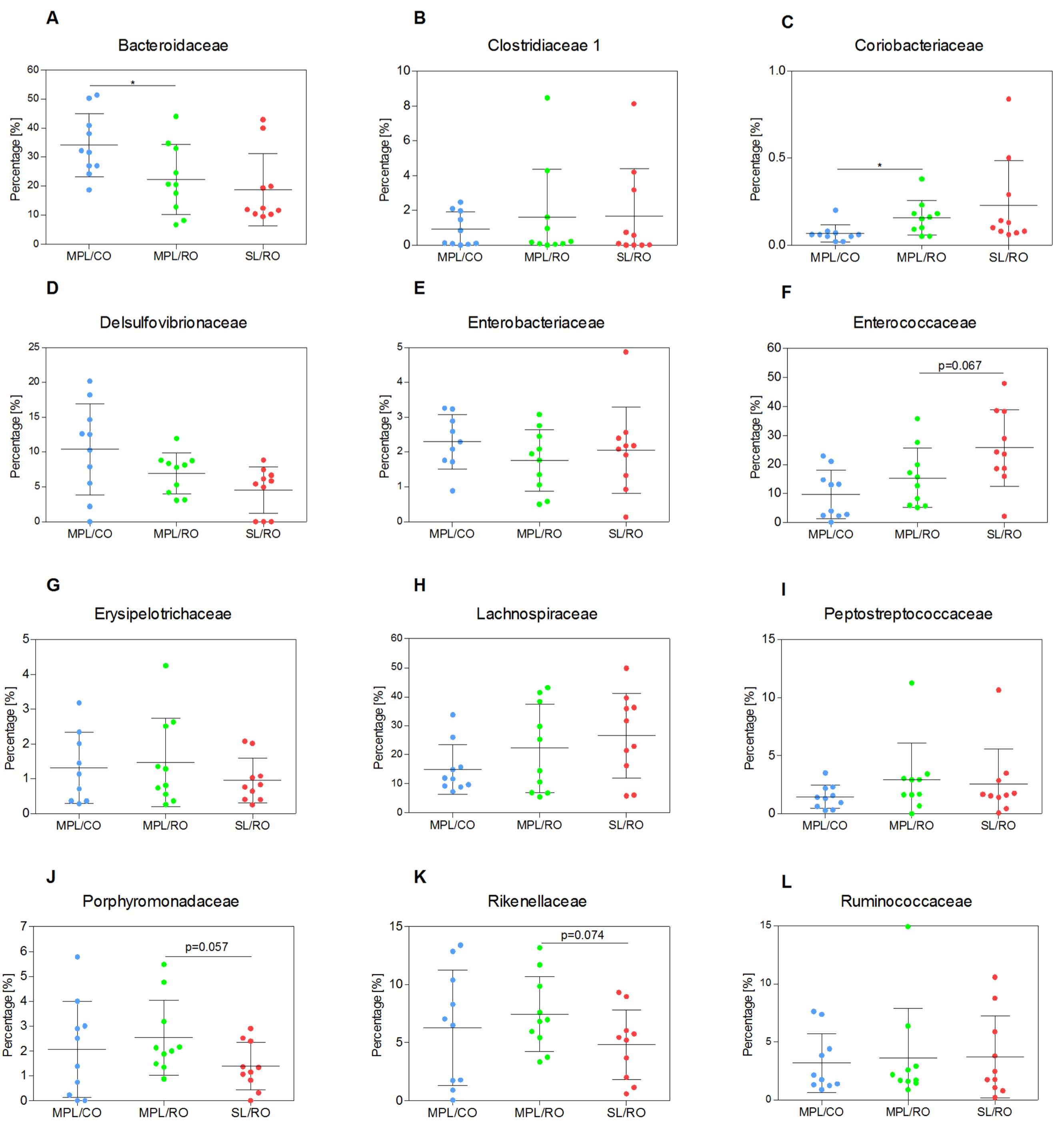

Figure S5 Percentage-vise bacterial distribution in cecum. Distributions are shown for A: Bacteroidaceae, B: Clostridiaceae 1, C: Coriobacteriaceae, D: Desulfovibrionaceae, E: Enterobacteriaceae, F: Enterococcaceae, G: Erysipelotrichaceae, H: Lachnospiraceae, I: Peptostreptococcaecae, J: Porphyromonadaceae, K: Rikenellaceae and L: Ruminococcaceae. Data are shown as percentage with mean±SD, n = 10. Number of asterisks indicate significant p-values (* $p<0.05$). No differences were found between groups within the same day. Any significant difference within groups over time is not illustrated. NB: Y-axes are not identical.

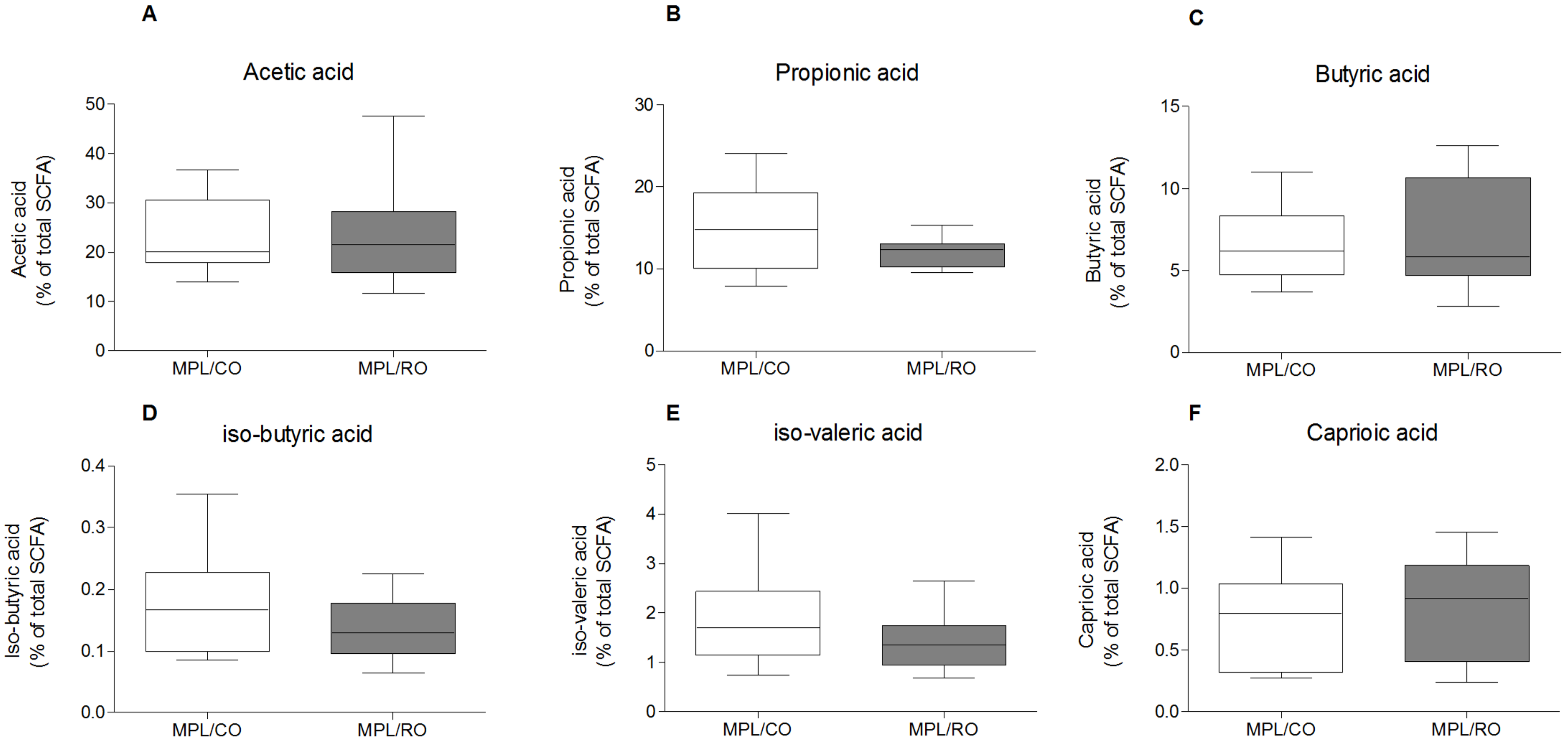

Figure S6 Effect of oil on short-chain fatty acid (SCFA) production in cecum. Levels of SCFAs are illustrated as percentage of total SCFAs. A: Acetic acid, B: Propionic acid, C: Butyric acid, D: iso-Valeric acid, E: iso-Butyric acid, and F: Caprioic acid. Differences between groups are calculated using student's t-test using a significance level of p=0.05. No differences were observed between groups.
